# Arrhythmogenesis in Fabry Disease

**DOI:** 10.1007/s11886-024-02053-2

**Published:** 2024-04-12

**Authors:** Ashwin Roy, Max J. Cumberland, Christopher O’Shea, Andrew Holmes, Manish Kalla, Katja Gehmlich, Tarekegn Geberhiwot, Richard P. Steeds

**Affiliations:** 1 Institute of Cardiovascular Sciences, University of Birmingham, Birmingham, UK; 2 Department of Cardiology, University Hospital Birmingham NHS Foundation Trust, Birmingham, Birmingham, UK; 3 Division of Cardiovascular Medicine, Department of Medicine and British Heart Foundation Centre of Research Excellence Oxford, University of Oxford, Oxford, UK; 4 Department of Inherited Metabolic Diseases, University Hospital Birmingham NHS Foundation Trust, Birmingham, Birmingham, UK; 5 Institute of Metabolism and System Research, University of Birmingham, Birmingham, UK

**Keywords:** Fabry, Arrhythmia, Atrial fibrillation, Sudden cardiac death

## Abstract

**Purpose of Review:**

Fabry Disease (FD) is a rare lysosomal storage disorder characterised by multiorgan accumulation of glycosphingolipid due to deficiency in the enzyme α-galactosidase A. Cardiac sphingolipid accumulation triggers various types of arrhythmias, predominantly ventricular arrhythmia, bradyarrhythmia, and atrial fibrillation. Arrhythmia is likely the primary contributor to FD mortality with sudden cardiac death, the most frequent cardiac mode of death. Traditionally FD was seen as a storage cardiomyopathy triggering left ventricular hypertrophy, diastolic dysfunction, and ultimately, systolic dysfunction in advanced disease. The purpose of this review is to outline the current evidence exploring novel mechanisms underlying the arrhythmia substrate.

**Recent Findings:**

There is growing evidence that FD cardiomyopathy is a primary arrhythmic disease with each stage of cardiomyopathy (accumulation, hypertrophy, inflammation, and fibrosis) contributing to the arrhythmia substrate via various intracellular, extracellular, and environmental mechanisms. It is therefore important to understand how these mechanisms contribute to an individual’s risk of arrhythmia in FD.

**Summary:**

In this review, we outline the epidemiology of arrhythmia, pathophysiology of arrhythmogenesis, risk stratification, and cardiac therapy in FD. We explore how advances in conventional cardiac investigations performed in FD patients including 12-lead electrocardiography, transthoracic echocardiography, and cardiac magnetic resonance imaging have enabled early detection of pro-arrhythmic substrate. This has allowed for appropriate risk stratification of FD patients. This paves the way for future work exploring the development of therapeutic initiatives and risk prediction models to reduce the burden of arrhythmia.

## Introduction

Fabry Disease (FD) in an X-linked inherited lysosomal storage disorder due to pathogenic variants in the galactosidase-α (*GLA*) gene, resulting in deficiency of the enzyme α-galactosidase A (α-GAL A) [1, 2]. This leads to widespread intracellular lysosomal accumulation of glycosphingolipids, namely, globotriaosylceramide (Gb3) and globotriaosylsphingosine (lyso-Gb3), in multiple organs. Accumulation causes intracellular and extracellular dysfunction by direct and indirect effects that lead to predominantly cardiac, renal, and cerebrovascular manifestations with significant associated morbidity and mortality [3].

Patients exhibit a spectrum of phenotypes depending on the mutation within the *GLA* gene, of which there are many. Nonsense, missense variants, and premature stop codons that lead to an (almost) complete loss of function of α-GAL A enzyme are mostly associated with classic, early-onset FD characterised by severe multi-organ disease beginning usually in childhood. Missense variants that reduce α-GAL A activity are mostly associated with predominantly single-organ involvement, particularly cardiac, presenting usually later in adult life. Due to the X-linked nature of the disease, it was presumed that heterozygous females were obligate carriers. However, it is now recognised that females exhibit similar degree of cardiac involvement, albeit later in life [4].

Cardiac sphingolipid accumulation takes place in all cardiac cell types, including cardiomyocytes, conduction system cells, fibroblasts, smooth muscle, and endothelial cells [5]. The classic feature of FD cardiomyopathy is the development of left ventricular hypertrophy (LVH), which is triggered by sphingolipid accumulation, but the mechanism is not understood. Traditionally, FD has been considered a simple storage cardiomyopathy in which LVH leads to diastolic and systolic left ventricular (LV) dysfunction, presenting with breathlessness and heart failure. There is, however, increasing evidence that arrhythmia and sudden cardiac death (SCD) may be more important. This review aims to outline types of arrhythmias, epidemiology of arrhythmia, and SCD in FD; summarise the evidence on pathogenesis; identify potential methods for risk stratification; and consider therapeutic options.

## Incidence and Prevalence of Arrhythmia

### Tachyarrhythmia and Sudden Cardiac Death

**Fig. 1:**
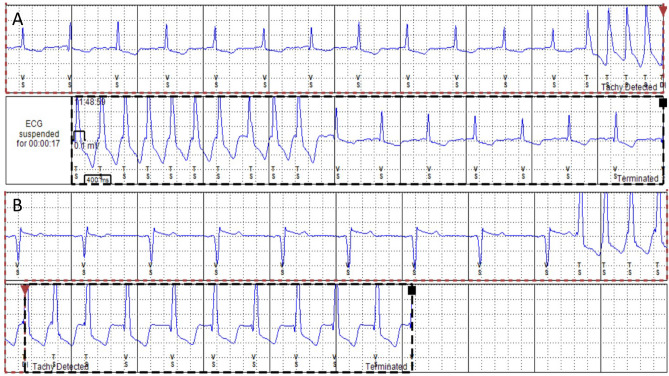
Non-sustained ventricular tachycardia (NSVT) detected on an implantable loop recorder (ILR) in a patient with FD

**Fig. 2:**
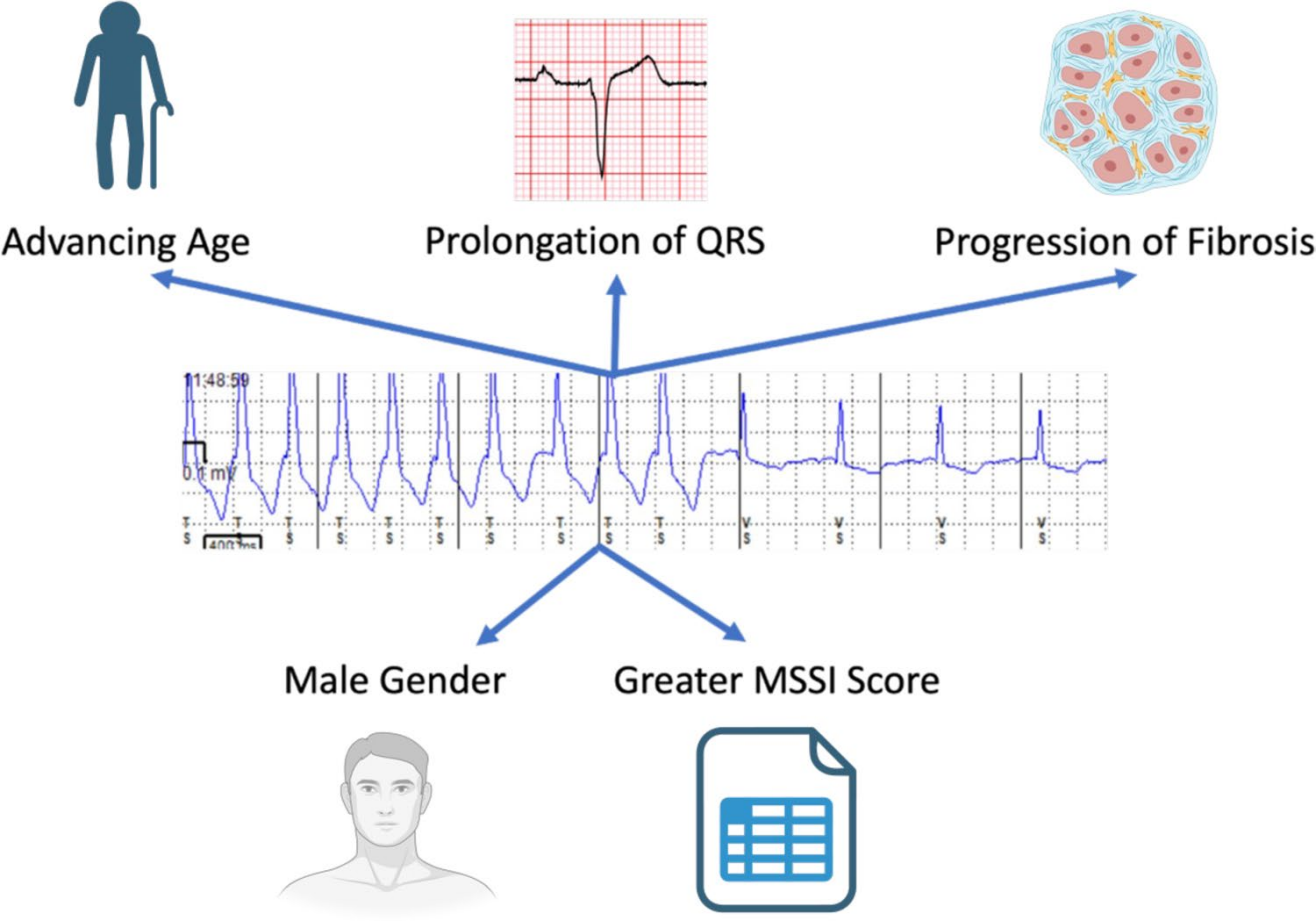
Risk factors for VA

Tachyarrhythmia is prevalent in FD and may be supraventricular or ventricular in origin. Atrial fibrillation (AF) is the most frequently reported supraventricular arrhythmia, and regular narrow complex tachycardias are less common, despite shortening of the PR interval [6]. Ventricular tachycardia (sustained and non-sustained) and ventricular fibrillation are the most frequent ventricular arrhythmias (VA), with overall prevalence between 13 and 18% [7–11]. Figure 1 illustrates the non-sustained ventricular tachycardia (NSVT) detected on an implanted loop recorder (ILR) in a patient with FD. The incidence of SCD varies between 0.5 and 3% (follow-up range 1.2–8 years) [7, 12, 13, 14•, 15••, 16], which is much higher than the rate in both the general population (0.03–0.1%) and patients with sarcomeric hypertrophic cardiomyopathy (0.5–1%) [17, 18]. Details of studies describing incidence and prevalence of VA and SCD are summarised in Table 1. Predictors of VA and SCD include advancing age, an annual increase in myocardial fibrosis, severity of FD measured by the Mainz Severity Score Index (MSSI), and prolongation of QRS duration on electrocardiogram (ECG) [8, 12]. Single-centre studies have demonstrated a higher prevalence of VA in males than females [7, 19]. Risk factors for VA are illustrated in Fig. 2. No change in frequency of tachyarrhythmia has been demonstrated solely according to mutation-type in FD [20].

**Table 1:** Study characteristics of incidence and prevalence of VA, ICD implantation, and SCD in Fabry Disease

Authors	Number of patients	Study type	Median follow-up	Type of monitoring	Incidence	Prevalence
Shah et al. (2005) [7]	78 baseline 66 follow-up	Single-centre longitudinal prospective	1.9 years	Holter	Sudden cardiac death: 1.5% ICD implantation: 1.5%	NSVT: 13%
Kramer et al. (2014) [8]	73	Single-centre retrospective observational	7.1 years	Holter		Malignant VA: 18% (39% of whom experienced sudden cardiac death)
Acharya et al. (2012) [9]	19	Single-centre retrospective observational	4.7 years	ECG		ICD implantation: 37% Indications: NSVT and syncope
Patel et al. (2015) [12]	207	Single-centre longitudinal prospective	7.1 years	ECG	Sudden cardiac death: 3%	
Weidemann et al. (2016) [13]	16	Multi-centre longitudinal prospective	1.2 years	ILR	ICD implantation: 25% Indication: malignant VA	
Orsborne et al. (2022) [14•]	200	Single-centre longitudinal prospective	4.5 years	Holter	Sudden cardiac death: 0.5% NSVT: 12%	
Meucci et al. (2023) [15••]	314	Multicentre retrospective observational	8 years	ECG	Cardiovascular mortality: 3.3% VA: 3.3%	
Vijapurapu et al. (2019 [16]	880	Multi-centre retrospective observational	4.3 years	ECG/cardiac device	ICD implantation: 4.8% Indications: symptomatic VA, NSVT, multiple risk factors and no arrhythmia, symptomatic long QT syndrome, pacemaker indication with NSVT NSVT: 17% in those with ICD Malignant VA requiring ATP/defibrillation: 28% of those with device	
Frustaci et al. (2015) [10]	13	Single-centre retrospective observational	N/A	ECG/Holter		VA: 15.3%
Deva et al. (2016) [11]	39	Single-centre retrospective observational	N/A	ECG/Holter		VA: 13%

### Bradyarrhythmia

**Fig. 3:**
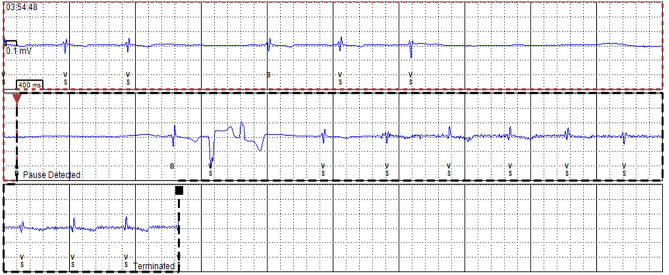
An asymptomatic daytime pause on ILR in a patient with FD

**Fig. 4:**
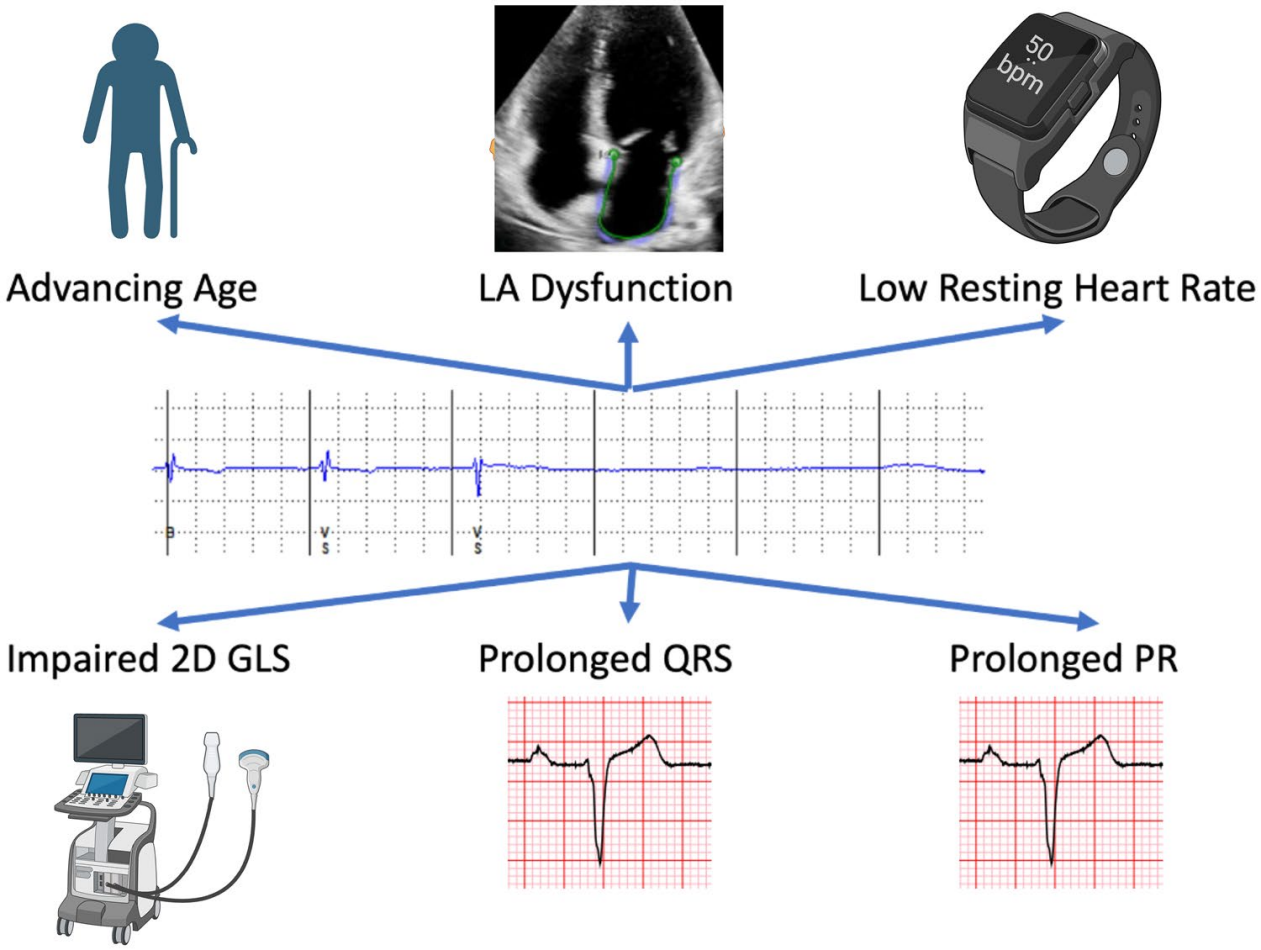
Risk factors for bradycardia

Bradyarrhythmia is more common than tachyarrhythmia in FD [21••]. In a recent systematic review, weighted estimates of event rate bradyarrhythmia were 10% (pooled median follow-up time 4.5 years) [21••]. Bradyarrhythmia that have been reported in FD patients include sinus node disease (specifically, symptomatic bradycardia, sinus pauses, and chronotropic incompetence) and atrioventricular (AV) nodal disease (including first-, second-, and third-degree AV block) [22]. In a study of 29 adults with FD (82% male; mean age 43 ± 11 years), the prevalence of resting bradycardia was reported as 72% (of which 7% had shortened PR interval, 7% prolonged PR interval, and 86% normal) [23], which may be attributed to early autonomic dysfunction and chronotropic incompetence. There is even data suggesting that sinus bradycardia may ensue in childhood with a study of 26 children with FD (46% male; mean age 9.7 ± 3.8 years) demonstrating that 23% had a resting bradycardia on 12-lead ECG [24]. Bradyarrhythmia relating to clinically significant sinus node disease (severe sinus bradycardia and sinus pauses) has been reported in up to 12% [25]. Bradyarrhythmia requiring permanent pacemaker implantation (PPM) varies between 2.5 and 11% (follow-up range 1.2–8 years) [7, 9, 12, 13, 16, 26–29]. Figure 3 illustrates an asymptomatic daytime pause on ILR in a patient with FD. Details of studies describing incidence and prevalence of bradyarrhythmia are summarised in Table 2. Risk factors associated with bradyarrhythmia include increasing age, left atrial (LA) dysfunction, lower resting heart rate, prolonged PR and QRS duration, use of beta-blocker therapy, and impaired LV global longitudinal strain (GLS) [25, 26, 28]. Risk factors for bradycardia are illustrated in Fig. 4. No changes in frequency of bradycardia have been demonstrated according to gender or mutation-type in FD [20].

**Table 2:** Study characteristics of incidence and prevalence PPM implantation due to bradycardia and AF in Fabry Disease

Authors	Number of patients	Study type	Median follow-up	Type of monitoring	Incidence	Prevalence
Shah et al. (2005) [7]	78 baseline 66 follow-up	Single-centre longitudinal prospective	1.9 years	Holter	Permanent pacemaker implantation: 10.6% Indications: complete heart block, symptomatic bradycardia, left ventricular outflow tract reduction Atrial fibrillation: 3%	Atrial fibrillation: 3.9% on ECG, 13% on Holter
Patel et al. (2015) [12]	207	Single-centre longitudinal prospective	7.1 years	ECG	Permanent pacemaker implantation: 6% Indications: not specified Atrial fibrillation: 6%	
O’Mahony et al. (2011) [26]	204 baseline 188 follow-up	Single-centre retrospective observational	4.8 years	ECG and Holter	Permanent pacemaker implantation: 6% Indications: atrioventricular nodal disease, sinus node disease	Permanent pacemaker implantation: 2.5% Indications: atrioventricular nodal disease, sinus node disease Atrial fibrillation: 3%
Weidemann et al. (2016) [13]	16	Multi-centre longitudinal prospective	1.2 years	ILR	Permanent pacemaker implantation: 19% Indications: asystole, symptomatic bradycardia Atrial fibrillation: 31%	
Di et al. (2018) [25]	53	Cross-sectional retrospective	N/A	ECG		Permanent pacemaker implantation: 11% Atrial fibrillation: 21%
Sene et al. (2016) [27]	49	Single-centre retrospective observational	8 years	ECG	Permanent pacemaker implantation: 12% Indications: sinus node dysfunction, atrioventricular disease	
Pichette et al. (2017) [28]	43	Single-centre retrospective observational	4.2 years	ECG	Atrial fibrillation: 13%	
Vijapurapu et al. (2019 [16]	880	Multi-centre retrospective observational	4.3 years	ECG/cardiac device	Permanent pacemaker implantation: 4.3% Indications: Tachy-Brady syndrome, sinus node dysfunction, bifascicular/trifascicular block, second/third-degree atrioventricular block AF: 19% (device-detected AF out of 90 with devices)	
Talbot et al. (2015) [29]	25	Single-centre retrospective observational	10 years	ECG	Permanent pacemaker implantation: 4% Indications: not specified	
Acharya et al. (2012) [9]	19	Single-centre retrospective observational	4.7 years	ECG	Atrial fibrillation: 10%	Permanent pacemaker implantation: 10.5% Indications: symptomatic bradycardia, conduction abnormalities
Meucci et al. (2023) [15••]	314	Multicentre retrospective observational	8 years	ECG	Bradyarrhythmia: 7.2% Atrial fibrillation: 7.6%	
Orsborne et al. (2022) [14]	200	Single-centre longitudinal prospective	4.5 years	Holter	Bradyarrhythmia requiring PPM: 3% Indications: not specified Atrial fibrillation: 4%

### Atrial Fibrillation

**Fig. 5:**
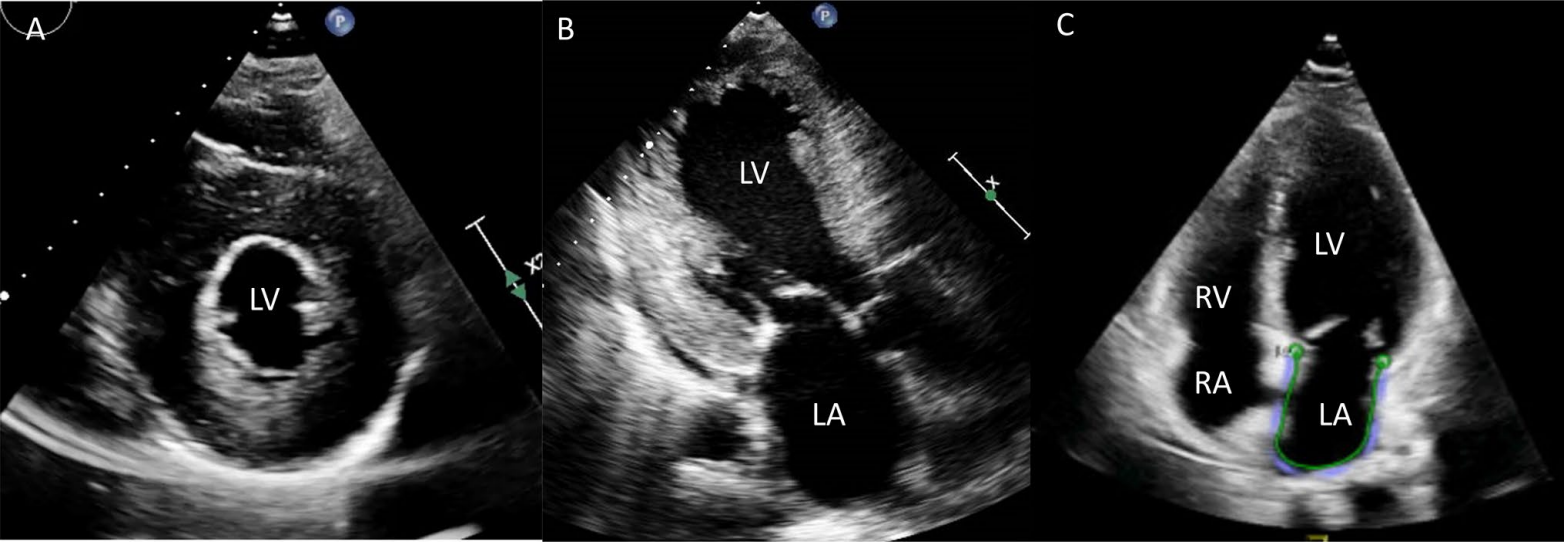
LVH raises left ventricular end-diastolic pressure which transmits to the LA, resulting in LA dilatation and impaired LA strain

AF is a supraventricular arrhythmia that involves irregular and disorganised atrial conduction arising due to electrophysiological disruption usually originating from the pulmonary veins. AF is prevalent in FD with reported figures between 3 and 21% [9, 25, 26]. Incidence of AF in FD from longitudinal data is reported between 3 and 31% (follow-up range 1.2–8 years) [7, 9, 12, 13, 16, 26–29]. The incidence is likely to be higher with numerous mechanisms in FD contributing to the structural, functional, and electrical substrate that is required for AF to initiate and persist. LVH raises left ventricular end-diastolic pressure which transmits to the LA, resulting in LA dilatation and impaired LA strain (see Fig. 5) [30]. LA strain impairment predicts new onset and recurrence of AF in the general population [31] and has also been found in patients with FD, with initiation of enzyme replacement therapy (ERT) improving function [32]. The same study demonstrated a higher incidence of AF and stroke in the cohort with impaired LA strain. Most of the patients had diastolic function without LVH suggesting that direct atrial sphingolipid accumulation may contribute to LA dilatation and impaired strain rather than passive effects of the LV [28].

### Limitations of the Data on Arrhythmia

The incidence and prevalence of both tachyarrhythmia, bradyarrhythmia, and AF are variably reported because the detection rate depends in part on the duration of monitoring. Data from small single-centre cohorts using continuous monitoring with advanced disease have detected a much higher rate of arrhythmia requiring intervention than studies using intermittent 24-h Holter monitoring [13, 33]. This is important because the annual Holter monitoring recommended by current guidelines is likely to under-estimate the frequency of arrhythmia [34]. The variation in reporting of arrhythmia is also likely to be attributable to differences in the cohorts studied, in terms of age, sex, genotype, and classical or non-classical phenotype. Studies suggest a greater number of cardiac events in males with classical FD compared with males with non-classical disease or females. However, these studies do not specify whether these relate specifically to arrhythmia [35]. Within these limitations, however, it is interesting to reflect on the possibility that FD patients seem to have a much higher risk of VA and SCD than sarcomeric hypertrophic cardiomyopathy, which is the paradigm for arrhythmogenesis and SCD [36]. Future studies using longer-term monitoring with implanted loop recorders have completed recruitment and outcomes are awaited [37].

## Pathogenesis

α-GAL A is a lysosomal enzyme which catalyses the cleavage of terminal galactose from glycosphingolipids. Absence or deficiency of α-GAL A leads to a failure to catabolise galactosyl moieties [38], which contribute in turn by direct and indirect effects on generation of arrhythmia.

*Direct Effects of* α*-GAL-A and Arrhythmia.*

Cardiac arrhythmias are common and can occur before other evidence of FD cardiomyopathy. Histology of myocardial biopsies containing elements of conduction tissue clearly demonstrated sphingolipid deposition that occurs both early and consistently in most FD patients [10]. This is supported by the fact that ECG abnormalities are one of the earliest signs of cardiac involvement in children and adults. Shortening of *P*-wave duration and QRS width indicative of accelerated depolarisation intervals have been demonstrated in FD patients without LVH. These patients also have QTc prolongation and dispersion suggesting increased repolarisation time [39]. Studies have demonstrated crista terminalis and AV nodal glycosphingolipid accumulation which may accelerate atrial conduction and account for shortening of the *P*-wave duration [40, 41]. Prolongation of QTc and QTc dispersion are known to predispose to life-threatening arrhythmia, usually ventricular in origin [42]. These changes reflect the direct effect of sphingolipid accumulation in conduction tissue, resulting in altered repolarisation time and dispersion that explains the occurrence of arrhythmia before the onset of LVH, LA dilatation, or impaired LV function. Advanced techniques in digital ECG analysis are increasingly recognised as improving our ability to detect cardiac involvement in FD at an earlier stage than changes on a 12-lead ECG alone [43].

### Primary Effects

**Fig. 6:**
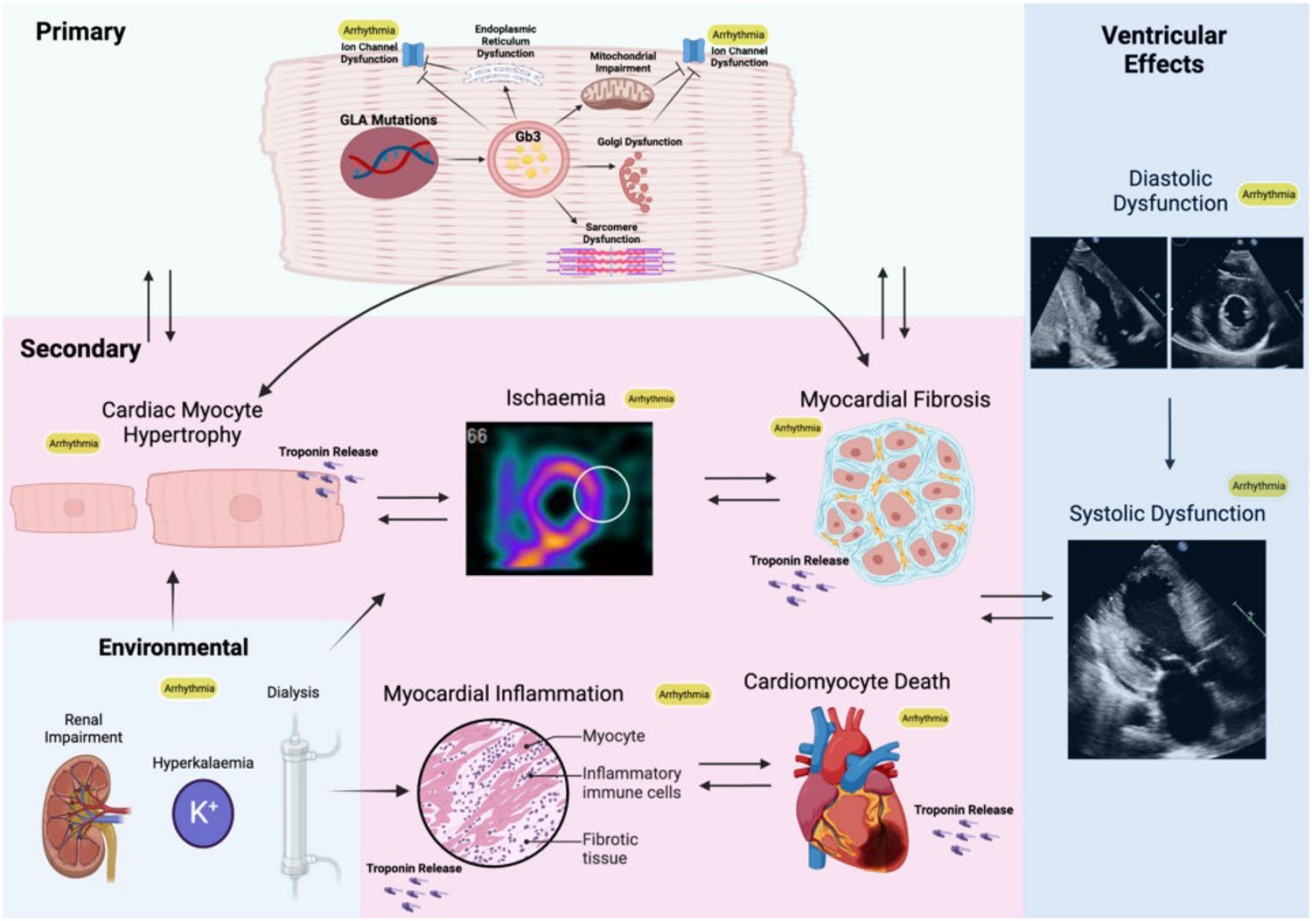
Pathophysiology of arrhythmia in Fabry Disease: Primary, secondary, and tertiary mechanisms

Whilst intracellular sphingolipid deposition within conduction tissue may have direct pro-arrhythmic effects, accumulation can trigger other deleterious processes either within the cell or because of ‘over-spill’ into the extracellular environment. Firstly, lysosomes are directly involved in cellular autophagy (Fig. 6). A key regulator of autophagy-lysosomal fusion and mitochondrial function is the mTOR-dependent signalling pathway [44]. mTOR is located on lysosomal surfaces and upon activation through amino acid accumulation, triggers autophagy [45]. Sphingolipid accumulation has been found to disrupt mTOR activation in FD cardiac fibroblasts and thereby inhibits autophagy-lysosome fusion [46]. mTOR is also involved in mitochondrial metabolism by promoting translation of mitochondrial-related proteins, which is adversely affected by lysosomal accumulation [47]. Reduced autophagy and impaired mitochondrial metabolism may play a vital role in inducing structural and functional changes within cardiac cells that could directly trigger arrhythmia [48]. Secondly, endomyocardial biopsies of FD patients demonstrate perinuclear vacuoles of glycosphingolipid. On electron microscopy, these vacuoles had the appearance of single-membrane-bound vesicles which displaced cardiac myofibrils to the periphery of the cell and induced myofibrillolysis. Cardiomyocyte area and percentage occupied by these vacuoles were similar, indicating that increased cardiomyocyte size may be explained by these vacuoles. The same study demonstrated that in FD patients with abnormal tissue Doppler indices on transthoracic echocardiography (TTE) indicative of diastolic dysfunction, in-vitro experiments on the cardiomyocytes of these patients also demonstrated stiffening [49]. Myofibrillolysis, vacuolation, and mitochondrial dysfunction may each contribute as trophic stimuli for hypertrophy [50], which forms a substrate for arrhythmia. Thirdly, data emerging from studies modelling FD in induced pluripotent stem cells (iPSC) have identified pro-arrhythmic intracellular mechanisms. A recent study characterised the structural and electrophysiological properties of FD patient-derived IPSC-derived ventricular cardiomyocytes with iPSCs obtained from patients with FD [51]. This study demonstrated that Fabry iPSC-derived cardiomyocytes had a higher spontaneous action potential (AP) frequency, shorter AP duration with an increased peak sodium current density, and upstroke velocity, suggesting increased excitability. Calcium transients had a greater amplitude and reduction in peak width duration, which indicate increased excitability and disruption to intracellular calcium handling. It has previously been shown that ion channel expression is affected by Gb3 accumulation in neuronal and endothelial cells [52, 53]. Therefore, α-GAL A deficiency and the accumulation of glycosphingolipid may alter the expression of calcium and sodium ion channels, causing severe effects on the electrophysiological properties of the cell. These early cellular changes likely cause an increased propensity for VA during disease development [51]. There is further scope using in-vivo and ex-vivo models for evaluation of arrhythmia used in combination in FD [54].

### Secondary Effects

**Fig. 7:**
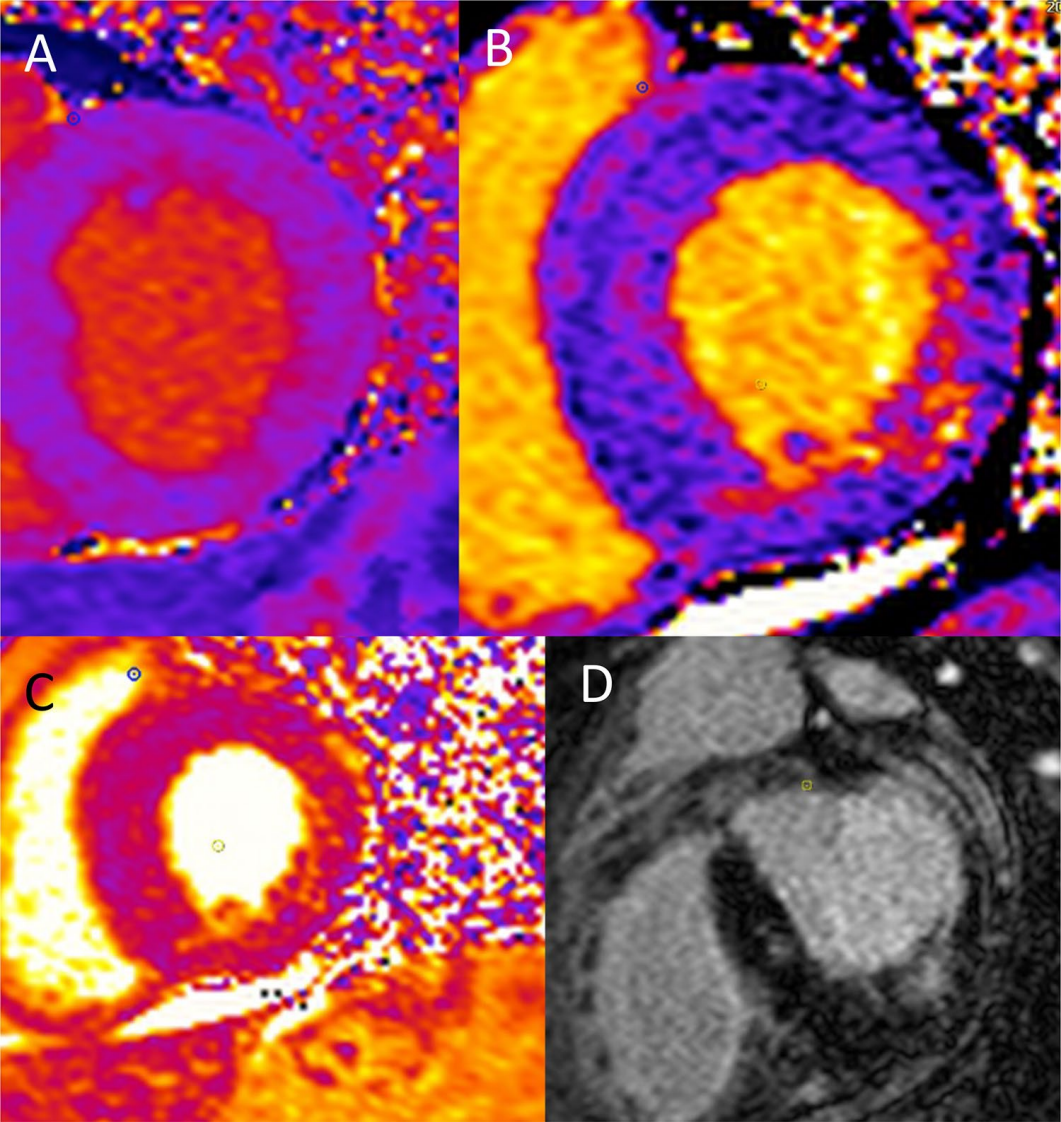
LGE identifies scar, predominantly in the basal inferolateral LV wall that can occur in women before development of hypertrophy, but seems only to develop in response to a hypertrophic stimulus in men
(ii) Inflammation and Arrhythmia
  Disorders of lysosomal storage are thought to have a significant effect on the immune system [60]. Lysosomal function involves maintenance of a normal immune system via antigen presentation, phagocytosis, and release of pro-inflammatory mediators [61, 62]. Specifically in FD, glycosphingolipid may cause damage-associated molecular pattern production which can induce cell apoptosis and pro-inflammatory cytokine secretion, which has been demonstrated when adding Gb3 to normal cells [63]. Leukocytes and endothelial cells in patients with FD also show signs of inflammatory activation, including generation of reactive oxygen species. A patient-specific inflammatory response may explain the significant intra-familial variability on clinical manifestations in FD. In a large study of adults with FD who underwent endomyocardial biopsy, histological evidence of myocarditis was demonstrated in 56% of patients with the presence of predominantly CD3 + T lymphocytes. Higher CD3 + T lymphocyte number correlated with increased wall thickness. In those without LVH, myocarditis was demonstrated in 38% of patients. The presence of myocarditis was associated with arrhythmia detected on Holter and ECG [64].
  Multiparametric cardiac MRI studies using LGE and T1/T2 mapping have enabled non-invasive detection and quantification of myocardial inflammation, consistent with these biopsy studies. Scar in the inferolateral wall detected on LGE imaging has conventionally been considered either to be replacement or reparative fibrosis. In FD however, elevated T2 scores that reflect oedema have been found not only to co-locate to the inferolateral scar on LGE (Fig. 7) but also correlate closely to increased high-sensitivity troponin and pro-inflammatory cytokines such as interleukin 6 [65, 66]. T2 elevation in the basal inferolateral wall suggestive of focal myocardial oedema has been associated with the ECG abnormalities that may provoke arrhythmia including abnormal PR interval, bundle branch block, and QTc prolongation [67]. That this oedema and troponin release is related to inflammation has been confirmed using 18-fludeoxyglucose (18-FDG) positron emission tomography (PET). In a study of 13 adults with FD, FDG-PET/cardiac MRI confirmed that focal LGE with increased T2 values corresponded to focal, increased 18-FDG uptake and associated high-sensitivity troponin elevation [68]. Moreover, in a study of female adults without LVH who underwent 18-FDG PET/cardiac MRI, increased focal 18-FDG myocardial uptake was detected in 54% of patients of whom only 15% had LGE on cardiac MRI. Uptake of 18-FDG uptake correlated with impaired LV GLS but in this ‘early group’, high-sensitivity troponin levels were normal. A further study in females with FD demonstrated an association between focal 18-FDG uptake and pseudonormalisation of T1 values, indicating this to be another marker of inflammation at the intermediate stage before the development of fibrosis associated with cell death [69]. These findings suggest that inflammation may be an insidious process that occurs before LVH and LGE and is not inevitably associated with myocyte cell death, so may be a modifiable target for therapy [70].
(iii) Multiorgan Involvement
  Renal sphingolipid accumulation is prevalent and prominent in podocytes [71]. This manifests as progressive renal dysfunction including microalbuminuria, proteinuria, and progressive decline in estimated glomerular filtration rate [72]. In advanced stages, FD patients may require long-term dialysis and assessment for renal transplantation [73]. Renal dysfunction predisposes to various arrhythmias including AF, bradyarrhythmia, VA, and SCD in the general population and its high prevalence in FD is likely to be a contributory mechanism to the arrhythmia substrate (see Fig. 6). The effects of electrolyte imbalance associated with renal dysfunction (namely potassium) as well as rapid fluctuation in electrolyte levels and haemodynamic shifts during dialysis further contributes to this increase in risk in the later stages of disease [74].

(i) Ischaemia, LVH, and Scar
  Cardiac sphingolipid accumulation only accounts for approximately 5% of the increase in ventricular mass in FD and is not associated with an increase in interstitial space [55]. Sphingolipid accumulation can be measured by detection of reduced T1 mapping times on cardiac magnetic resonance imaging (MRI) and is known to precede the development of LVH. How this triggers myocyte hypertrophy is not known. Of interest, smooth muscle cell accumulation within myocardial vasculature initiates structural and functional changes that cause microvascular and macrovascular ischaemia (Fig. 6). Stress perfusion mapping by cardiac MRI confirmed ischaemia in the absence of epicardial coronary stenosis in FD and showed this pre-dated the development of LVH in the affected myocardium [56]. FD is also characterised by the development of interstitial myocardial fibrosis, quantified by volume of extravascular, extracellular deposition of gadolinium contrast on late gadolinium enhancement (LGE) on cardiac MRI [57, 58]. LGE identifies scar, predominantly in the basal inferolateral LV wall (see Fig. 7), that can occur in women before development of hypertrophy, but seems only to develop in response to a hypertrophic stimulus in men [59]. Ischaemia, LVH, and interstitial scar are established factors in both inherited cardiomyopathies and acquired disease for arrhythmogenesis.

## Risk Stratification

Given the susceptibility to arrhythmia in patients with FD across all spectrums of the disease, risk stratification is imperative to establish those who would benefit from therapy. In the case of sarcomeric hypertrophic cardiomyopathy (HCM) there is a validated and universally accepted prediction model to evaluate individualised risk of SCD [75]. Those in a high-risk category are offered a primary-prevention implantable cardioverter defibrillator (ICD) to reduce the risk of VA and SCD. No such tool is available in FD on which to base decisions on therapy. To date, there have been single-centre studies and systematic reviews confirming risk factors for arrhythmia discussed so far, including advancing age, male gender, prior arrhythmia, LVH, and scar on LGE [21••, 76]. One important note is that, once patients have a risk of one type of arrhythmia, the change in substrate that takes place in FD seems to place the individual at increased risk of any arrythmia. For example, in a study evaluating device implantation in FD, one patient with a PPM died from sustained VF detected on device interrogation [16]. This suggests that, given the progressive nature of FD cardiomyopathy, considerable caution should be taken on implanting cardiac devices without a defibrillator function even in the absence of VA. In the absence of an individual risk calculator, there have been two recent large studies that used deep cardiac phenotyping to develop risk models in FD, with arrhythmia a major component of cardiovascular events recorded [14•, 15••].

A longitudinal prospective cohort study of 200 adults (average age 46 years; 61% female) with FD undergoing cardiac MRI developed a prognostic model to generate risk estimates for major adverse cardiac events (MACE) over a median follow-up of 4.5 years [14•]. MACE outcomes, based on time to first event, included hospitalisation for heart failure, myocardial infarction, coronary revascularisation, VT, new onset AF, bradycardia requiring implantation of a PPM, aborted SCD, implantation of an ICD, or cardiovascular death. A new parameter was developed from the cardiac MRI data, T1 dispersion, calculated as a standard deviation of T1 times extracted from all voxels consisting of the basal and mid-ventricular short-axis T1 maps in the mid-wall of the myocardium. In this study, the composite outcome occurred in 43 participants (21% total), at an annualised rate of 4.8% per year, and the most frequent component of the composite outcome was NSVT. The rate of VA was 0.44% per year, AF 1.67% per year, and bradycardia requiring pacemaker implantation 1.11% per year. Pooled univariable Cox regression for time to the composite outcome was performed, and variables with the highest statistic were age, maximum wall thickness (MWT), indexed LV mass, LV GLS, QRS duration, and MSSI. An internally validated model was developed which accurately predicted the 5-year risk of MACE using age, indexed LV mass, and native non-contrast T1 dispersion in all adults with FD.

In a recently published retrospective observational multi-centre study of 314 adults with FD, patients were staged on the degree of FD cardiomyopathy using TTE-based parameters [15••]. Study endpoints were used to test the prognostic value of the staging classification. Endpoints included all-cause mortality, cardiac mortality, major arrhythmia (bradyarrhythmia requiring PPM or tachyarrhythmia cardioversion/ICD therapy), new onset AF, heart failure hospitalisation, and ischaemic stroke. Patients were classified as stage 0 (no cardiac involvement), stage 1 (LVH with MWT > 12 mm), stage 2 (LA enlargement with indexed LAl volume > 34 ml/m^2^), or stage 3 (ventricular impairment). Older patients had more advanced disease and the majority with AF or cardiac device implantation were in stages 2 and 3. In total, 18% of patients met a study endpoint. Occurrence of cardiac events increased with stage and arrhythmic events accounted for 45% of these over the 8-year follow-up, with event rates for bradyarrhythmia 7.2%, VA 3.3%, and new onset AF 7.6% with a higher prevalence in more advanced stages. Interestingly, the stage with the highest frequency of PR duration abnormalities was stage 0, consistent with the known early electrical changes. Importantly, the role of LA dilatation and dysfunction as a parameter for prognosticating is highlighted here. LA dilatation may reflect elevated left ventricular end-diastolic pressure from LVH but dilatation may pre-date LVH secondary to direct atrial myopathy [77]. A greater proportion of female patients were in stage 2 (atrial enlargement) compared with males. Conversely, a greater proportion of male patients were in stage 1 (LVH), highlighting the varied pathophysiology and arrhythmic risk based on gender observed in FD [15•]. This study highlights how a greater burden of cardiac involvement correlates with cardiac events but with the large number of endpoints forming the composite (with arrhythmia included); this precludes accurate arrhythmic prediction.

Various risk-prediction data provide conclusive evidence of arrhythmic risk in other cardiomyopathies including sarcomeric HCM, lamin A/C, dilated cardiomyopathy, cardiac sarcoid, and cardiac amyloid [75, 78–81]. These enable clinicians to assess an individual’s risk of arrhythmia using conventional investigations including 12-lead ECG, Holter, TTE, and CMR. The existing risk prediction studies in FD provide more detailed information confirming that arrythmia is common but that other major adverse cardiovascular events traditionally included in cardiovascular outcome trials, including hospitalisation for heart failure, myocardial infarction, and cardiovascular death, are less frequent and follow-up time needs to be over longer periods. For arrhythmia, which clearly represent a significant burden to the patient in terms of quality of life, further work is needed to define individual risk in the way that the HCM-SCD risk calculator performs [75]. A prospective multi-centre international randomised control trial is currently underway in adults with FD comparing the rate of significant arrhythmia using ILRs [37]. This trial will also collect data from a wide variety of investigations with the aim to develop a robust risk stratification tool in FD.

## Therapy

### Conventional Risk Modification

Prevalence of conventional risk factors for atherosclerosis in FD is higher than in the general population suggesting accelerated atherosclerosis [82]. Interestingly, novel mechanisms of atherosclerotic disease have also been shown specifically in FD suggesting accelerated atherosclerosis via non-conventional mechanisms [82]. Although there is no randomised evidence of benefit specifically in FD, these data highlight the importance of aggressive risk factor modification including lipid-lowering therapy, antihypertensive therapy, and strict glycaemic control. Lifestyle modification including smoking cessation and regular physical exercise is also recommended [83]. Elevated systolic blood pressure has an incremental impact on progression of cardiomyopathy and subsequent arrhythmic risk due to relation with myocardial hypertrophy and geometry [84]. The benefits of angiotensin-converting enzyme (ACE) inhibitors and angiotensin receptor blocker (ARB) therapy in reducing cardiovascular risk have been demonstrated in sarcomeric HCM [85].

### Arrhythmia

Patients with FD tolerate arrhythmia supraventricular tachycardia and AF poorly due to impaired diastolic filling. Therefore, rhythm control is the favoured strategy with either pharmacological intervention, direct current cardioversion (DCCV), or ablation, although long-term success is compromised by progressive atrial dilatation, impaired atrial function, and change in atrial structure. Although there is now general support for early ablation in all patients presenting with AF, there remain concerns regarding long-term efficacy in FD [86]. In those with refractory AF not amenable to cardioversion, careful rate control is important, with the additional concern regarding co-existing risk of bradyarrhythmia and conduction disease. Beta blockers or calcium channel antagonists are generally favoured as first line for rate-limiting strategies, although amiodarone is generally avoided long term in FD. Amiodarone may alter lysosomal pH, subsequent enzyme activity, and trigger lysosomal dysfunction. It has also been shown to induce phospholipidosis via inhibition of lysosomal phospholipase activity, triggering phospholipid accumulation and development of lamellar bodies [87]. Amiodarone may induce acute heart failure decompensation with features of amiodarone toxicity confirmed on endomyocardial biopsy in a case study of amiodarone initiation in FD [88]. Frequent Holter or wearable ECG monitoring is recommended.

Although anticoagulation is recommended for life in all FD patients with a history of AF due to the high incidence of stroke, none of the current scoring systems for assessing risk of stroke, such as the CHA_2_DS_2_VAS_c_ scoring system [89], have been validated for use in FD. There is little systematic data on choice of anticoagulation (direct oral anticoagulant versus warfarin). However, direct oral anticoagulation may be advantageous due to lower risks of intracranial bleeding, as FD may be associated with cerebral micro-bleeding [90].

As described, cardiac device implantation with PPM or ICD is high due to increased risk of bradyarrhythmia and VA, particularly in advanced disease [21, 76]. However, with no specific guide for device implantation in FD currently available and as FD is specifically excluded from conventional risk calculators for HCM for primary prevention, device implantation is generally for secondary prevention after a significant arrhythmic event or aborted SCD [83].

### Enzyme Replacement Therapy

The mainstay of systemic FD-related therapy is ERT (recombinant α-GAL A) to replace deficient α-GAL A. Current recommendations are that in adult males with classical variants and enzyme activity < 5%, FD therapy is to be considered at diagnosis. In adult females and males with non-classical variants, FD therapy is recommended in those with an LV wall thickness > 13 mm in males and > 12 mm in females, indexed LV mass on TTE/CMR above normal for age and sex or the presence of LGE on CMR [34]. As with all FD therapy, better outcomes are reported with early initiation. There is limited efficacy if commenced > 50 years of age in terms of stabilising LV mass compared to if started < 30 years of age [72]. Those without LGE have seen improvements in LV mass, strain, and exercise capacity with ERT, which is not observed in those with LGE [91]. Efficacy in FD cardiomyopathy is mixed. Once many of the cardinal features of FD cardiomyopathy have developed, which increase arrhythmic risk, the benefits of ERT initiation are significantly limited. There is currently no definitive evidence that ERT reduces the burden of arrhythmia in FD [72, 92].

### Oral Chaperone Therapy

Oral chaperone therapy (OCT) is a pharmacological chaperone licensed for use in adults with FD with residual enzyme and an amenable *GLA* mutation. The mechanism is to stabilise the mutant enzyme, increase bioavailability, and traffic this to the lysosome whereby metabolism of sphingolipid can take place [93]. As with ERT, efficacy of OCT in established cardiomyopathy is limited and there is no evidence demonstrating the benefit of OCT on reducing arrhythmia burden. OCT may slow organ damage with studies showing a stabilisation and, in some cases, mild reduction in LVMi [94, 95]. However, in those with evidence of LGE, cardiomyopathy still progresses even with OCT [96].

### Transplantation

In patients with significant symptomatic heart failure, intractable arrhythmia despite all optimal therapy (medical and cardiac devices including synchronisation), transplantation may be a viable option. The major barrier to cardiac transplantation in FD is the progressive and multi-organ nature of the disease. Patients often have co-existing end-organ renal disease and so combined cardiac and renal transplantation may need to be considered. The involvement of multiple organs and increased disease burden has a significant impact on exercise capacity and mental health which affects transplant candidacy [97]. Finally, in patients with non-classical disease including cardiac variants, these are often late-onset in age which presents another barrier to transplantation [98].

## Conclusion

Mechanisms underpinning susceptibility for arrhythmia in FD are vast. Electrical instability begins early in the disease. Inflammation, previously presumed a more advanced process, also begins early and is a significant contributor to the arrhythmia substrate. Understanding the molecular mechanisms will enable better targeted therapy to reduce the burden of arrhythmia and SCD in this at-risk cohort. The development of a robust FD-specific risk-stratification tool will enable at-risk patients with FD to be identified and offered primary prevention cardiac device therapy. Further work is essential in these domains to improve cardiac outcomes in FD.

## Data Availability

Not applicable as this review did not involve the collection of data.
